# Why test for proportional hazards—or any other model assumptions?

**DOI:** 10.1093/aje/kwae002

**Published:** 2024-02-06

**Authors:** Arvid Sjölander, Paul W Dickman

The Cox proportional hazards model is a common analysis tool for survival data. In a recent tutorial, Stensrud and Hernán^1^ (SH) argued that hazards are rarely proportional in medical studies, and they concluded that tests for proportional hazards are “unnecessary.” They did not appear to argue against the Cox proportional hazards model per se, but asserted that the estimated hazard ratio from the model should be interpreted as a weighted average over time-specific hazard ratios.

Although we agree in principle, we find SH’s narrow focus on the Cox proportional hazards model potentially misleading, since it may give the false impression that this particular model is uniquely unrealistic or problematic for medical research. We are also concerned that readers may interpret the statement that “tests for proportional hazards are unnecessary”^1^^,p.1402^ to mean that one does not have to assess the appropriateness of the proportional hazards assumption at all. Our concerns can be summarized into the following points, upon which we elaborate below. The first point is somewhat trivial, whereas the second may be more controversial.

1. It is indeed unnecessary to test whether the proportional hazards assumption holds *exactly*, but the same can be said about any other statistical model assumption.
2. It is important to assess whether the proportional hazards assumption holds *approximately*, and there may often be no obvious a priori reasons to rule this out.

## Point 1

Most inferential statistical analyses are based on assumptions. Some of these are often emphasized, such as the proportional hazards assumption in Cox regression or the assumption of normality and homoscedasticity (constant variance) in linear regression, whereas others are typically given less attention, such as the assumption of independent and identically distributed observations. The proportional hazards assumption can be viewed as a type of no-interaction assumption; specifically, it assumes the absence of statistical interaction (on the hazard ratio scale) between the treatment and the underlying time scale in the model. When SH claimed it is “unnecessary” to test for proportional hazards, they presumably meant that it is unnecessary to test whether this assumption holds *exactly*, since we know a priori that it does not. This is true, but the same can be said about all other model assumptions that we make, since *all models are wrong, to some extent*. No hazards are perfectly proportional, no variables have perfectly normal distributions, no statistical interactions are perfectly absent, and—strictly speaking—no observations are perfectly independent (eg, because all humans, mice, etc, are biologically related on some level). In small- to moderate-sized samples, statistical tests may fail to reject such model assumptions due to a lack of power. However, since all models are to some extent wrong, all model assumptions will be rejected if the sample is large enough. In this trivial sense, it is “unnecessary” to test any statistical model assumption.

## Point 2

Does this mean that we should not care about assessing model assumptions? No! What it means is that, if we use a statistical model, we need to verify that the model assumptions hold *approximately*, so that the model gives reasonably accurate inference. This point was made succinctly by George Box, who stated in a famous quote that “all models are wrong, but some are useful.”^2^ Similar points have been made many times since then in the statistical literature.^3^^,^^4^

Whether a particular model fits sufficiently well to be useful is primarily an empirical question, which can be assessed with model diagnostic tools in the context of subject matter knowledge and the research question. For the Cox proportional hazards model, several diagnostic tools exist, mainly based on different kinds of residuals.^5^

In some scenarios, the underlying (eg, biological) mechanisms for the treatment and the outcome may also cast serious doubt on the proportional hazards assumption, thus endangering the usefulness of the model. SH considered 3 such mechanisms: no immediate effect of the treatment, immediate and delayed effects in opposite directions, and variations in disease susceptibility. The first 2 mechanisms imply clinically important deviations from the proportional hazards assumption, which make the Cox proportional hazards model inappropriate. There are certainly studies where one may suspect that either of these 2 mechanisms are present; however, we believe that one would more often suspect that the treatment effect has the same sign for the whole (or most of the) follow-up.

In contrast, variations in disease susceptibility will always be present. Thus, it is important to understand the implications for the hazard ratio. SH stated that “[e]ven if [the treatment] increased the risk of disease by a constant factor (eg, by 80%) at every single time of follow-up, it is still possible that the hazard ratio would have declined from 1.8 during the first year of follow-up to less than 1 in later years because the most susceptible women would have been diagnosed with [the] disease in the early follow-up.”^1^^,p.1401^ This is true, but the argument neglects the possibility that both the treatment effect and the disease susceptibility are nonconstant. For instance, suppose that the treatment increases the risk of disease by 80% at the beginning of follow-up and by 90% at the end of follow-up. Then, this increase in treatment effect may “cancel out” the depletion of susceptible women, so that the hazard ratio remains constant (eg, equal to 1.8) during follow-up. In Appendix S1, we give a numerical example of how this can happen.

In practice, one would rarely expect that the hazards are *exactly* proportional due to a *perfect* cancellation of a nonconstant treatment effect and a depletion of susceptible subjects. However, this is not necessary for the Cox proportional hazards model to be appropriate. Rather, to use the model we should require that the hazards are approximately proportional, and we see no obvious a priori reason why this could not be achieved in many studies by an approximate cancellation.

We are *not* claiming that hazards are approximately proportional in all, or even most, medical studies. We are also *not* claiming that the Cox proportional hazards model can be justified by stating, as we do here, that there are “no obvious a priori reasons” why hazards couldn’t be (approximately) proportional. What we do claim is that SH’s arguments against (approximate) proportionality are not very strong, and thus do not generally disqualify the Cox proportional hazards model from medical research. As we stated above, the validity of the model is mainly an empirical question, which in our experience is highly context-dependent; for some data the model does fit reasonably well, and for other data it does not.

## An analogy with logistic regression

Some may feel that the aforementioned cancellation of a nonconstant treatment effect and a depletion of susceptible subjects is unrealistic, even if only approximate. In response, we point out that the Cox proportional hazards model is not unique in this respect; other common regression models require similar artifacts. For instance, consider the standard logistic regression model

$$\mathrm{logit}\ p=\mathrm{\alpha}+\mathrm{\beta} X,$$

where $p$ is the risk of the disease and $X$ is a set of measured predictors. Whatever predicators we have measured and conditioned on, there will always be residual variation in disease susceptibility, due to unmeasured factors $U$ that influence the risk of the disease. In order to obtain the logistic regression model above when “averaging” over $U$, one would have to require an extreme fine-tuning of the parameters in the “true” (given both $X$ and $U$), possibly complex, model. In Appendix S1, we elaborate on this issue and provide a detailed example.

We are *not* attempting to justify the Cox proportional hazards model by arguing that the standard logistic regression model is also a priori unrealistic. Indeed, in our view neither model is a priori utterly unrealistic. Our point is rather that, if one accepts SH’s arguments against the Cox proportional hazards model, it seems like one has to dismiss many other common regression models as well, via similar arguments. We don’t know whether SH agree with this point, or whether they are as skeptical about logistic regression as they are about Cox proportional hazards regression. However, their narrow focus on the Cox proportional hazards model gives the impression that they view this model as uniquely unrealistic and problematic, for which we see no obvious reason.

## Conclusion

Statistical inference is built upon assumptions. While we note that not all assumptions are equally realistic and not all assumptions are necessary for inference, we also note that the proportional hazards assumption is similar to other assumptions commonly made in statistical modeling. Formal statistical tests of proportional hazards may be unnecessary, but analysts should assess the appropriateness of the assumption for their data and research question. Thus, analysts must understand the assumption, how and why it might be violated, and how one interprets estimated hazard ratios from a proportional hazards model. The tutorial by SH is an excellent resource for gaining such understanding.

If the Cox proportional hazards model (or any other simple regression model) is deemed inappropriate in a given situation, we encourage the analyst to use more sophisticated analysis tools that require less strict assumptions—for example, machine learning methods.^6^

## Supplementary material

Supplementary material is available at *American Journal of Epidemiology* online.

## Funding

This work was funded by Swedish Research Council grant 2020-01188 (A.S.).

## Conflict of interest

The authors declare no conflicts.

## Supplementary Material

Web_Material_kwae002
