# Supplementary material for: Why test for proportional hazards—or any other model assumptions?
Source: Am J Epidemiol. 2024 Feb 6;193(6):926–7. doi: 10.1093/aje/kwae002 (PMC11579528; doi:10.1093/aje/kwae002)
Supplement: Web_Material_kwae002 [file web_material_kwae002.pdf]

SUPPLEMENTARY MATERIAL

Why test for proportional hazards – or any other model assumptions?

Arvid Sjölander, PhD, and Paul Dickman, PhD

Appendix S1

Perfect cancellation in a Cox proportional hazards model

Let X be a binary randomized treatment. Let follow-up be discretized into two time points, $t = 1,2$, and let Y_t be the binary indicator of the outcome happening at time t, for $t = 1,2$. Let U be the whole set of (unmeasured) factors that influence both Y_1 and Y_2. The causal diagram in Figure S1 illustrates the situation. Note that Y_2 is only measured if $Y_1 = 0$, i.e., if the subject survived $t = 1$.

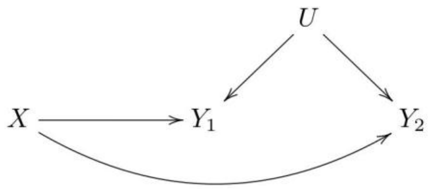

Figure S1: Causal diagram.

The discrete-time hazard ratios at $t = 1$ and $t = 2$ are given by

$$HR_1 = \frac{p(Y_1 = 1|X = 1)}{p(Y_1 = 1|X = 0)}$$

and

$$HR_2 = \frac{p(Y_2 = 1|Y_1 = 0, X = 1)}{p(Y_2 = 1|Y_1 = 0, X = 0)}.$$

Suppose that U is binary; we may think about subjects with $U = 1$ as "more susceptible for the outcome" and subjects with $U = 0$ as "less susceptible for the outcome". Define $q = p(U = 1), r = p(X = 1), p^1_{xu} = p(Y_1 = 1|X = x, U = u)$ and $p^2_{xu} = p(Y_2 = 1|Y_1 = 0, X = x, U = u)$. Using the fact that X and U are marginally independent we can now write

$$\begin{aligned} p(U = 1|Y_1 = 0) &= \frac{p(Y_1 = 0|U = 1)q}{p(Y_1 = 0|U = 1)q + p(Y_1 = 0|U = 0)(1 - q)} \\ &= \frac{\{(1 - p^1_{01})(1 - r) + (1 - p^1_{11})r\}q}{\{(1 - p^1_{01})(1 - r) + (1 - p^1_{11})r\}q + \{(1 - p^1_{00})(1 - r) + (1 - p^1_{10})r\}(1 - q)}, \end{aligned}$$

$$p(U = 1|Y_1 = 0, X = x) = \frac{(1 - p^1_{x1})q}{(1 - p^1_{x1})q + (1 - p^1_{x0})(1 - q)},$$

$$HR_1 = \frac{p_{10}^1(1-q) + p_{11}^1 q}{p_{00}^1(1-q) + p_{01}^1 q}$$

and

$$HR_2 = \frac{p_{10}^2 p(U=0|Y_1=0, X=1) + p_{11}^2 p(U=1|Y_1=0, X=1)}{p_{00}^2 p(U=0|Y_1=0, X=0) + p_{01}^2 p(U=1|Y_1=0, X=0)}$$
$$= \frac{p_{10}^2(1-p_{10}^1)(1-q) + p_{11}^2(1-p_{11}^1)q}{p_{00}^2(1-p_{00}^1)(1-q) + p_{01}^2(1-p_{01}^1)q} \times \frac{(1-p_{00}^1)(1-q) + (1-p_{01}^1)q}{(1-p_{10}^1)(1-q) + (1-p_{11}^1)q}.$$

Suppose now that $q = r = 1/2$, $p_{00}^1 = 1/4$, $p_{01}^1 = p_{10}^1 = 1/2$, $p_{11}^1 = 3/4$, $p_{00}^2 = 1/3$, $p_{01}^2 = p_{10}^2 = 1/2$, $p_{11}^2 = 1$. For these figures, the prevalence of $U = 1$ at $t = 2$ is $p(U = 1|Y_1 = 0) = 3/8$. This is less than the baseline prevalence $q = 1/2$, so there has indeed been a depletion of subjects susceptible for the outcome during follow-up. Furthermore, the conditional treatment effect, given U, is not constant over follow-up; for $U = 0$ we have that

$$\frac{p_{10}^1}{p_{00}^1} = 2 \neq \frac{p_{10}^2}{p_{00}^2} = 3/2$$

and for $U = 1$ we have that

$$\frac{p_{11}^1}{p_{01}^1} = 3/2 \neq \frac{p_{11}^2}{p_{01}^2} = 2.$$

Nevertheless, $HR_1 = HR_2 = 5/3$, so that the proportional hazards assumption holds.

Perfect cancellation in a logistic regression model

Let Y be a binary outcome and let X and U be two non-binary predictors for Y. For simplicity, we assume that X and U are independent. To illustrate our point about “fine-tuning” of parameters, suppose that the conditional probability of $Y = 1$, given X and U, is given by the logistic regression model

$$logit\{p(Y = 1|X, U)\} = \alpha^* + \beta^* X + \gamma^* U + \psi^* XU. \quad (1)$$

Under this model, the conditional probability of $Y = 1$, given X only, is obtained by integrating over the marginal distribution of U:

$$logit\{p(Y = 1|X)\} = logit\left\{\int p(Y = 1|X, U = u)p(u)du\right\}$$
$$= logit\left[\int expit\{\alpha^* + \beta^* X + \gamma^* u + \psi^* Xu\} p(u)\, du\right].$$

This expression generally depends on the predictor X in a complicated way, and does not generally simplify to the marginal (over U) model

$$logit\{p(Y = 1|X)\} = \alpha + \beta X, \quad (2)$$

except possibly for very specific (“fine-tuned”) values of the parameters $(\alpha^*, \beta^*, \gamma^*, \psi^*)$ in combination with a very specific parametric distribution $p(u)$. One such fine-tuning is when ψ^* is exactly 0 and $p(u)$ is a bridge distribution.[1]

In this example, we considered the particular conditional model in (1); however, a similar problem would arise if we would consider other forms of conditional models, e.g., a probit regression model, or a model with splines or other types of nonlinearities.

References

1. Wang Z, Louis TA. Matching conditional and marginal shapes in binary random intercept models using a bridge distribution function. *Biometrika* 2003;90(4):765-775.
